# Human papillomavirus 16 infection in adenocarcinoma of the cervix

**DOI:** 10.1038/sj.bjc.6602855

**Published:** 2005-11-01

**Authors:** G K Chew, M E Cruickshank, P H Rooney, I D Miller, D E Parkin, G I Murray

**Affiliations:** 1 Department of Gynaecology Oncology, Aberdeen Royal Infirmary, Foresterhill, Aberdeen AB25 2ZD, UK; 2 Department of Medicine and Therapeutics, University of Aberdeen, Foresterhill, Aberdeen AB25 2ZD, UK; 3 Department of Pathology, University of Aberdeen, Foresterhill, Aberdeen AB25 2ZD, UK

**Keywords:** HPV, adenocarcinoma, cervix, real-time PCR, laser capture microdissection

## Abstract

The impact of the success of organised cervical screening programme results in a steady decline of the incidence of squamous cell carcinoma of the cervix but a concomitant increase in the incidence of the less common histological subtypes, particularly adenocarcinoma of the cervix (ACC). Although Human papillomavirus (HPV) infection is believed to be a necessary cause of cervical cancer, its role in the pathogenesis of ACC is not well established. Established associations between oncogenic strains of HPV and ACC are based on molecular studies carried out on entire tumour block sections. In this study, the cervical adenocarcinoma cells of a 10-year cohort of women diagnosed with ACC were dissected using the PixCell II Laser Microdissecting System to detect the HPV 16 genome sequence using the real-time quantitative polymerase chain reaction to confirm the presence of HPV DNA within ACC cells. By coupling these two sophisticated techniques, the HPV DNA copy number cell could be calculated to investigate its role. The prevalence of HPV 16 infection in this cohort was 24%, which is significantly higher than the control group (*χ*^2^, *P*=0.014). Women with ACC also had significantly higher HPV DNA copy number per cell compared to the control group (*P*=0.00007). Higher HPV DNA copy number is associated with risk of developing ACC.

In countries such as the United Kingdom, organised cervical screening has resulted in a steady decline in the overall incidence of cervical cancer. Cervical screening has enabled identification and treatment of squamous epithelial changes in its pre-malignant state, leading to a noted decline in the incidence of squamous cell carcinoma (SCC) in all age groups. Conversely, there is a concomitant rise in the incidence of ACC. This is largely due to a relative increase, but there is evidence of an absolute increase in the incidence of ACC in more recent birth cohorts (Sasieni and Adams, 2001); particularly in women aged less than 35 years old.

HPV infection is necessary for the development of cervical cancer (Walboomers *et al*, 1999). While the role of HPV in the pathogenesis of ACC is less well understood, the association of HPV infection in ACC and in antecedent cervical cytology have been reported (Xavier-Bosch *et al*, 1995). HPV infection is highly prevalent and is usually transient. It is effectively cleared by the immune system 90% of the time, suggesting that other biological, environmental and viral-specific factors must trigger the malignant transformation.

HPV infection has a defined role in the aetiology of squamous intra-epithelial changes. The incidence of concurrent intra-epithelial changes in the presence of ACC is quoted as high as 40% (Maier and Norris, 1980). In a study where the presence of the HPV subtypes were identified using immunohistochemical expression of the p53 gene product, HPV16 infection was found in the adjacent uninvolved cervical squamous epithelium (Ferguson *et al*, 1998). It is therefore possible that the incidence of HPV infection in ACC has been exaggerated in studies where HPV infection was identified in DNA extracted from sections of tumour blocks.

A possible solution would be to employ the sophisticated technology afforded by the laser capture microdissecting (LCM) system, which selects specific cell of interest for analysis, with minimal loss and destruction of DNA material (Curran *et al*, 2000). In this study, we coupled two sophisticated mechanisms of LCM and real-time quantitative PCR (RTqPCR) to define the presence and amount of HPV16 DNA in cervical adenocarcinoma cells.

## MATERIALS AND METHODS

### Chemicals

PCR grade Proteinase K digest is purchased from Roche. The Tween 20 (Polyxyethylene Sorbitane) and Ethylenediamine tetraacetic acid (EDTA) were purchased from Sigma. The DNA extraction buffer comprised a mixture of 50 mM Tris/HCl (pH8.5), 1 mM EDTA. The Nucleon BACC II DNA extraction kit used was from Tepnel Life Sciences, Manchester, UK. The TAQMAN Universal PCR Mastermix, Passive Reference and optimised buffer components was from Applied Biosystems, CA, USA.

### Ethics

We obtained permission from the Grampian Research Ethics Committee to identify the cases and controls from the Grampian University Hospitals (GUH) pathology database and to use archival paraffin-fixed specimens for analysis.

### Case and control samples

Using the GUH population-based pathology database, a cohort of women diagnosed with ACC between 1991 and 2000 were identified. The archival paraffin-embedded tissue blocks were retrieved from the GUH tissue bank and reviewed by two consultant pathologists to confirm the histopathological diagnoses.

Age-matched controls were identified from women who had undergone hysterectomy or amputation of cervix in 1991–2000. Four controls were selected for each case by year of operation and their date of birth were matched to within 24 months of their case.

The tissue sections were prepared for laser microdissection as follows: 5 *μ*m sections were cut onto glass slides and dried overnight. The sections were dewaxed and stained with 0.25% Touloudine blue (pH4.5). The cells were microdissected using the Pixcell II Laser Microdissection System (Arcturus Engineering, CA, USA) using the following settings: laser pulse spot 15 *μ*m, voltage 100 mW and duration of laser exposure 10.0 milliseconds. Approximately 500 pulses are taken per tumour sample.

The adenocarcinoma cells were dissected and used for the cases. For the controls, the columnar epithelium was microdissected to avoid contamination of possible HPV infection in the squamous epithelium. The squamous epithelial cells were laser microdissected and the DNA extracted from these cells to analyse for the HPV 16 gene sequence.

The transfer film containing the dissected cells was removed from the vial cap (CapSure™ HS, Arcturus Engineering, CA, USA) and incubated in a proteinase K digest (Tween, Proteinase K and digest buffer) on a heating block at 55°C and then heat inactivated at 98°C. DNA extracted from these cells was stored at 4°C for sequence analysis.

### Laser capture microdissection system

The Pixcell II Laser Microdissection System (Arcturus Engineering, CA, USA) is an inverted microscope with an infrared dissection laser, which targets the sample from above. The cells are transferred onto a transfer film, which is permanently bonded to the underside of a transparent vial cap (CapSure™ HS).

### Real-time quantitative PCR

The ABI 7700 PRISM® Sequence Detector (Applied Biosystems, CA, USA) enables real-time quantitative PCR (RTqPCR) analysis for particular gene sequences to allow quantitation of PCR product.

### Probes and primers

The primer and probe sets for RTqPCR were designed using the Primer Express 1.0 software. The complete genome sequences of HPV16 (accession number NC 001526) and *Beta-globin* (accession number NG 000007.2) gene were entered into the programme to design primer and probe sets optimal for the ABI7700 PRISM® sequence detector (Applied Biosystems, CA, USA). A BLAST search was performed (http://www.ncbi.nlm.nih.gov/BLAST) for each primer and probe oligonucleotide sequence to confirm specificity for the target locus. The probe was labelled with the fluorescent reporter molecule *FAM,* which emits as the PCR product is formed. The specific primer and probe sets were ordered from Applied Biosystems. The Beta-globin primer and probe sets had been used successfully with the ABI7700 Sequence Detector (Rooney, 2005).

### CaSki cell line growth condition

The CaSki cell line was cultured in Dulbecco's medium (Life Technologies) supplemented with 10% (v/v) fetal calf serum (FCS) in a 37°C incubator with 5% CO_2_/95% air with media changes twice weekly. Cultured cells were passaged weekly using PBS (Oxoid), followed by detachment of the cells using a mixture of trypsin/EDTA and then incubated at 37°C for 5 min. The cells are pelleted and seeded into sterile flasks where they were washed with ice-cold PBS and scraped into 10 ml ice-cold PBS. 100 *μ*l of the cell suspension is removed and added to 100 *μ*l of Trypan buffer. Once the cells were counted using a haemocytometer, the suspension was centrifuged to give a pellet.

### Real-time quantitative PCR

The DNA templates were assessed for HPV16 gene copy by real-time quantitative PCR using the ABI7700 sequence detector (Applied Biosystems, CA, USA). *Beta-globin* gene sequence was used as a reference gene, the positive PCR product confirmed successful DNA extraction. A no-template triplicate was used as a negative control. DNA extracted from CaSki cell line pellet using the Nucleon BACC II method (Tepnel Life Sciences, Manchester, UK) was used as positive control. Each CaSki cell contains 600 copies of the HPV16 gene (Meissner, 1999).

**Figure 1:**
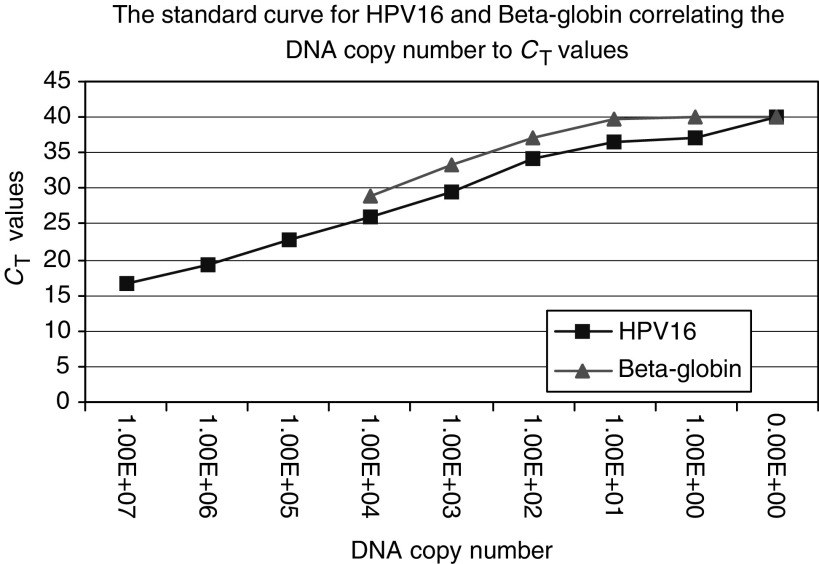
Standard curve for HPV 16 and *Beta-globin*.

A standard curve was generated for HPV 16 and *Beta-globin* DNA copy numbers using the average *C*_T_ generated by quantitative PCR analysis of serially diluted DNA extract of a known numbers of Caski cells (Figure 1). Each sample of DNA template was analysed in triplicate for both *Beta-globin* and HPV16 DNA sequence for 40 cycles. The average *C*_T_ for HPV16 and *Beta-globin* of each sample were plotted, respectively, on the standard curve to calculate the DNA copy number. The HPV 16 DNA copy per cell is calculated by the ratio of the HPV 16 to *Beta-globin* copy number.

### Classification of HPV 16 DNA copy number

In this study, DNA copy number of <100 copies per cell was classified as low HPV 16 DNA copy number. DNA copy number of ⩾100 copies per cell as high HPV16 DNA copy number (Table 1).

### Statistical analysis

The 2 × 2 *χ*^2^ tests were used to evaluate the risk of HPV 16 infection and the HPV 16 DNA copy number on the risk of developing ACC. A probability value of <0.05 was considered significant. Statistical analysis was carried out using the EpiInfo 6.04 b January 1997 package (A word Processing Database and Statistics Programme for Public Health, Centre for Disease Control and Prevention and World Health Organisation, Geneva, Switzerland).

## RESULTS

**Figure 2:**
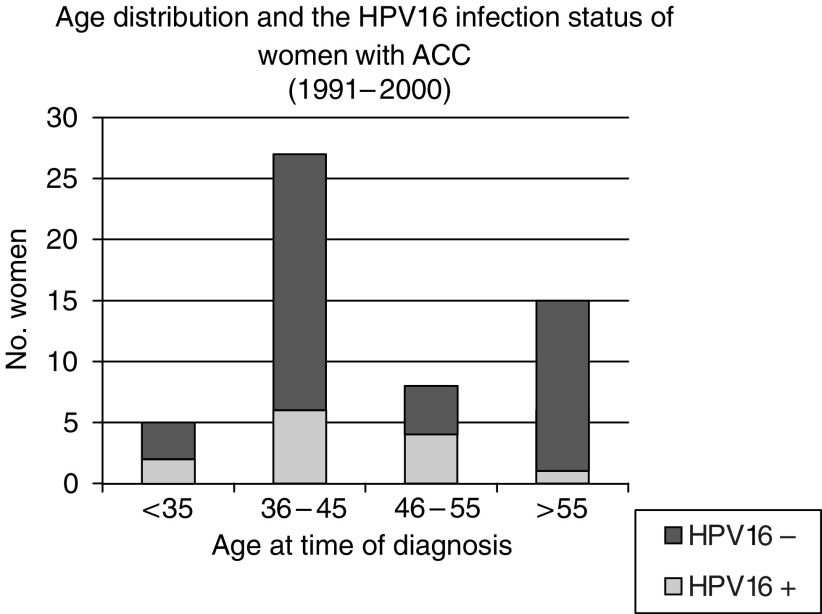
The age distribution of women with ACC (1991–2000). The shaded areas represent HPV 16 positive women.

A total of 55 cases of ACC were identified from the GUH pathology population-based database. The age distribution showed a bimodal pattern, with peaks in the 36–45 and over 55 years age groups (Figure 2). In all, 82% of the ACC were well or moderately differentiated cancer (Table 2). In all, 208 controls were identified.

Successful DNA extraction, defined by presence of *Beta-globin* gene sequence, was found in all the samples. Real-time quantitative PCR analysis showed that 24% (13/55) of the cases were HPV 16 positive. In the control group, 21/208 (10%) were positive for HPV16. HPV 16 infection was 2.7 times more likely to be found in ACC cells than in the control group and this finding is statistically significant (Table 3). (*χ*^2^ *P*=0.014; Yates – corrected) Odds Ratio=2.76 (95% CI 1.19–6.33).

In HPV 16 positive ACC cases, 77% had low DNA copy numbers. All the HPV 16 positive controls had low DNA copy numbers. The HPV 16 DNA copy number per cell is significantly higher in the HPV 16 positive ACC cells compared to the controls (see Table 4) (*χ*^2^ *P*=0.00007). There was no statistical difference in the HPV copy number in the grade of the cancer (Table 2). The HPV 16 DNA copy number is significantly higher in women over 45 years old (*P*=0.03) (Table 5).

The squamous epithelium adjacent to the cervical adenocarcinoma cells was analysed separately for HPV 16 infection. In all, 39 specimens had squamous epithelium in the archival specimens; the remaining 16 samples were punch biopsies of the endocervix and had no adjacent squamous epithelium in the archival specimens. In all, 10 of the 39 cases with adjacent squamous epithelium for analysis, had HPV 16 in the adenocarcinoma cells. Seven squamous epithelium specimens were positive for HPV infection. Of this, three had HPV 16 infection in both squamous and columnar epithelium. All three had low HPV 16 DNA copy number in the squamous epithelium. All three women were over 40 years old at the time of their diagnosis. Although the frequency of simultaneous infection is higher, the association is not statistically significant.

## DISCUSSION

The key finding in this study is the direct demonstration of HPV 16 DNA within the cervical adenocarcinoma cells. It confirmed the infection of the adenocarcinoma cells and therefore allowed the demonstration of the true prevalence of HPV16 infection in ACC. This added significant strength to the link between HPV 16 infection and the aetiology of adenocarcinoma of the cervix. The demonstration of HPV 16 infection in the adjacent squamous epithelium but not in the adenocarcinoma cells, supports our hypothesis that the previously recorded high prevalence of HPV 16 infection in ACC is due to infection in adjacent squamous epithelium.

We have shown that using the LCM technique of tissue selection for analysis, a higher yield of DNA extraction is achieved. The specificity is also increased as we can confirm that the HPV 16 DNA is found within the tumour cells of ACC. Contamination of HPV 16 DNA in concurrent CIN can also be avoided. We also decrease the false negative rates by reducing the loss by dilution effect. By extracting DNA only from the tumour cells, the amplified PCR product has a higher concentration of HPV DNA.

Previously published studies were based on a qualitative analysis of presence or absence of the PCR product. By using real-time quantitative PCR, the cycle by cycle detection of accumulated PCR product allows us to generate a standard curve, in order to calculate the gene DNA copy number. In conjunction with the LCM, for the first time, we are able to expand our understanding of HPV infection in ACC by studying the effect of HPV DNA viral load on the cancer cells.

Women with ACC are 2.7 times more likely to have HPV 16 infection compared to the control population and have a higher HPV DNA copy number than the controls. The prevalence of HPV 16 infection in our control population is 10%, which is consistent with epidemiological studies and all of these women had a low HPV 16 DNA copy number. This finding strongly supports the theory that most HPV16 infection is transient (Schiffman, 1992) and higher HPV DNA copy number is associated with development of cervical carcinoma (Ylitalo *et al*, 2000).

Over half of the HPV 16 positive samples came from women under 45 years old, which reflects the sexually transmissible nature of the infection. By using real-time quantitative PCR, we have demonstrated the variation in HPV 16 copy number across this population. The DNA copy number, however, is higher in women over 45 years old. One possible explanation for this finding is DNA replication during the longer incubation period of HPV16 infection before transformation to malignancy (Stanley, 2001). The lower HPV DNA copy number in the women under 45 years old may be explained by infection of multiple HPV subtypes or infection by high risk HPV 16 subtypes.

Direct infection of columnar cells is the main route of HPV 16 infection, as only 27% of the HPV 16 positive cases have concurrent infected squamous epithelium. This lower prevalence on concurrent HPV infection in the squamous epithelium can be explained by clearance of HPV infection from the squamous epithelium. In Stanley's immunobiological model on HPV infection of squamous epithelium, HPV infection is targeted at the basal epithelial cells. It is possible that the infected basal cells, of Stanley's biological model, could have differentiated into glandular epithelial cells and therefore the squamous cell will bear no evidence of HPV infection.

By coupling the two powerful techniques of laser capture microdissection and real-time quantitative polymerase chain reaction, we were able to demonstrate a more accurate picture of the role of HPV infection in the pathogenesis of cervical adenocarcinoma.

## Figures and Tables

**Table 1:** Classification of HPV 16 DNA copy number

**DNA copy number**	
**Low count**	**High count**
0.1	100
1	1000
10	

**Table 2:** The HPV DNA count in the well, moderately or poorly differentiated Adenocarcinoma of the cervix

	**Low HPV16**	**High HPV 16**	**HPV 16 neg**
Well	6	1	13
Moderate	3	2	19
Poor	1	0	7
Total	10	3	39

**Table 3:** HPV 16 in ACC cases and age-matched controls

	**Cases**	**Controls**
HPV 16 +	13	21
HPV 16 −	42	187

*P*=0.014 (*χ*^2^; Yates – corrected) Odds Ratio=2.76 (1.2 <OR <6.3) 95% CI 1.19–6.33.

**Table 4:** HPV 16 copy number in cases and controls

	**ACC cases**	**Age matched controls**
High HPV 16 DNA copy no.	4	0
Low HPV 16 DNA copy no.	9	21

(*χ*^2^test) *P*=0.00007.

**Table 5:** HPV 16 DNA copy number according to the woman's age at time of diagnosis

**Age**	**<35**	**36–45**	**46–55**	**>55**
HPV16 −	3	21	4	14
HPV16 (low)	2	6	2	0
HPV16 (high)	0	0	2	1

Fisher 1-tailed *χ*^2^ *P*=0.03; RR=5; 1.45<RR <17.27.
